# Novel insulin receptor substrate 1 and 2 variants in breast and colorectal cancer

**DOI:** 10.3892/or.2013.2626

**Published:** 2013-07-18

**Authors:** DIANA LIBERATA ESPOSITO, FABIO VERGINELLI, SONIA TORACCHIO, SANDRA MAMMARELLA, LAURA DE LELLIS, CINZIA VANNI, ANTONIO RUSSO, RENATO MARIANI-COSTANTINI, ALESSANDRO CAMA

**Affiliations:** 1 Unit of General Pathology, Aging Research Center, G. d’Annunzio University Foundation, I-66100 Chieti, Italy; 2 Department of Medical, Oral and Biotechnological Sciences, University G. d’Annunzio Chieti-Pescara, I-66100 Chieti, Italy; 3 Department of Pharmacy, University G. d’Annunzio Chieti-Pescara, I-66100 Chieti, Italy; 4 Hospital SS Annunziata, Laboratory of Clinical Pathology, I-66100 Chieti, Italy; 5 University of Palermo, Department of Surgical and Oncological Sciences, I-90133 Palermo, Italy

**Keywords:** breast cancer, colorectal cancer, insulin receptor substrate 1, insulin receptor substrate 2

## Abstract

The insulin/insulin-like growth factor pathway is involved in breast and colorectal cancer (CRC) development. In the present study, we analyzed the coding region and short intron-exon borders of the *insulin receptor substrate 1* and *2* (*IRS-1* and *IRS-2*) genes in 12 cell lines derived from breast cancer (BC), 14 cell lines derived from CRC and 33 primary CRCs. The nucleotide variants identified in BC were 3 in IRS-1, 1 of which (p.Arg267Cys) was novel and with a pathogenic potential as predicted by *in silico* analysis and 6 in IRS-2. Twenty-one variants in IRS-1 and 18 in IRS-2 were identified in the CRC samples. These included 11 novel IRS-1 variants detected exclusively in CRCs, which included 5 missense (p.Pro559Leu, p.Gln655His, p.Asp1014Gly, p.Asp1181His and pPro1203Ser) with a pathogenic potential as predicted by *in silico* analysis, 2 frameshifts predicted to generate a truncated protein, 1 splice-site mutation and 3 silent variants. In the CRC samples we also identified 7 novel IRS-2 variants, including 4 missense variants, which included 2 (p.Asp782Asn and p.Gly1230Ser) with a pathogenic potential as predicted by *in silico* analysis, 2 frame insertion mutations and 1 silent variant. Most of the novel IRS-1 and IRS-2 variants may be involved in the modulation of IRS-1 or IRS-2 functions and could be relevant to breast and colorectal tumorigenesis.

## Introduction

Insulin, insulin-like growth factor 1 and 2 (IGF-1 and IGF-2) and IGF binding protein (IGFBP) are involved in cell growth and survival and are thought to be implicated in colorectal cancer (CRC). The insulin receptor substrates (IRS) are cytoplasmic signaling adaptor proteins that function as intermediates of the insulin receptor (IR) and IGF-IR (1). In addition, IRS proteins signal downstream of integrin, cytokine and steroid hormone receptors (2,3). By mediating the activities of these receptors, the IRS proteins play a central role in maintaining diverse cellular functions, such as metabolism, motility, survival and proliferation. Four IRS proteins have been described. Considering that IRS-3 is expressed only in rodents (4) and IRS-4 shows limited tissue expression (brain, kidney, thymus and liver) (5), most studies have been focused on IRS-1 and IRS-2, both of which are widely expressed. Tyrosine-phosphorylated IRS-1/-2 bind proteins containing Src homology 2 (SH2) domains, such as the p85 regulatory subunit of the PI3K, the phosphotyrosine phosphatase SHP-2, the Src-like kinases Fyn, Grb-2, NCK, CRK, SHB and others (6). These activate downstream effector cascades, such as the mitogen-activated protein kinase (MAPK) and the PI3K pathways which promote biological responses (6). Irs1^−/−^ mice display glucose intolerance, but do not develop overt diabetes (7). Irs2^−/−^ mice have been shown to develop diabetes as a consequence of decreased β-cell function and insulin resistance (8). Therefore, IRS-1 and IRS-2 possess both similar and distinct properties.

The human *IRS-1* gene (*IRS-1* OMIM: 147545), spanning 64 kb of genomic DNA (gDNA) on chromosome 2q36, comprises 1 exon and encodes an 8743-bp mRNA while the human *IRS-2* gene (*IRS-2* OMIM: 600797), spanning 32.732 kb of gDNA on chromosome 13q34, comprises 2 exons and encodes a 7014-bp mRNA.

Polymorphisms of IRS-1 (G972R) and IRS-2 (G1057D) have been independently associated with CRC risk (9). Moreover, IRS-1 G972R significantly modifies the risk of developing ovarian cancer in BRCA1 and BRCA2 mutation carriers (10). Our previous results suggest that IRS-1 may influence adenoma formation, CRC progression and liver metastasis (11). Expression of IRS-1 can be directly activated by β-catenin, likely in part via β-catenin/TCF binding to TCF consensus binding elements located in the first intron and downstream of the IRS-1 transcriptional start site (12). Moreover, one study showed that partial or absolute IRS-1 deficiency reduces the tumor load in APC^min/+^ mice (13). IRS-2 was reported to be amplified in 3 out of 146 primary CRCs (14). Therefore IRS-1 and IRS-2 are most likely implicated in CRC and breast cancer (BC). For these reasons, we analyzed human primary CRC tumors and cell lines for genetic variants in the coding regions of the *IRS-1* and *IRS-2* genes. *IRS-1* and *IRS-2* coding regions were also analyzed in BC cell lines.

## Materials and methods

### Cell lines and CRC patients

DNA was analyzed in the following CRC cell lines: CaCo2, CBS, Colo205, DLD1, HCT15, HCT116, HT29, Int407, LoVo, Mip101, SW480, SW620, WiDr, Geo and cell lines derived from BC: BT-20, BT-474, EVSA/T, MCF10A, MCF7, MDA-MB-134, MDA-MB-231, MDA-MB-365, MDA-MB-453, SK-BR-3, T47D, HCC1937, obtained from Professor Stefano Iacobelli, University of Chieti, Italy and Professor Maurizio Alimandi University ‘La Sapienza’, Rome, Italy. Moreover, we analyzed 33 sporadic frozen CRCs collected at the Department of Oncology, University of Palermo, Palermo, Italy. Additionally, 60 formalin-fixed/paraffin-embedded (FFPE) CRCs showing microsatellite instability-high (MSI-H) as previously described (15,16) were studied for 2 IRS-1 genetic alterations, c.119delG and c.1791delG. Collection and analysis of samples were approved by the G. d’Annunzio University Ethics Committee.

In the case of IRS-2, the control sequences included those obtained by Bottomley *et al*(17) in 173 normal subjects. Furthermore, we performed IRS-2 mutational analysis in 25 control subjects (50 alleles), and the screening was extended to more alleles for some variants. The variants identified in the present study were verified in the NCBI database of single nucleotide polymorphisms (IRS-1, http://www.ncbi.nlm.nih.gov/projects/SNP/snp_ref.cgi?geneId=3667; IRS-2, http://www.ncbi.nlm.nih.gov/projects/SNP/snp_ref.cgi?geneId=8660) and in the 1,000 genome database (IRS-1, http://browser.1000genomes.org/Homo_sapiens/Transcript/ProtVariations?db=core;g=ENSG00000169047;r=2:227308182-227372719;t=ENST00000305123; IRS-2, http://browser.1000genomes.org/Homo_sapiens/Transcript/ProtVariations?db=core;g=ENSG00000185950;r=13:109204185-109236916;t=ENST00000375856).

### DNA extraction

DNAs from the cell lines (5×10^6^ cells) were isolated by QIAamp DNA Blood Mini kit (Qiagen GmbH, Hilden, Germany) according to the manufacturer’s protocol. Frozen CRCs were snap-frozen in optimal cutting temperature (OCT) medium. Multiple cryosections from each OCT block were collected onto glass slides and fixed with 70% ethanol. Sections were microdissected, and gDNA was extracted by QIAamp DNA Tissue Mini kit (Qiagen GmbH) according to the manufacturer’s protocol and using three 15-μm sections for each tumor. For frozen samples and formalin-fixed CRCs an area with at least 50% neoplastic cells and an area including normal muscularis propria and/or CRC-unaffected mucosa were identified on H&E-stained slides and used to guide manual microdissection for DNA extraction. Serial sections 15-μm thick were prepared for DNA extraction. Selected areas were dissected from de-waxed step-sections by gentle scraping. Scraped tissue was digested by incubation overnight at 56°C in 100 ml of buffer containing Tris (50 mM pH 8.5), EDTA (1 mM), Tween-20 (0.5%) and proteinase K (20 mg/ml). The extracted DNA was purified with the QIAamp DNA Mini kit following manufacturer’s instructions.

### Mutational analysis

The coding region and short intron-exon borders of IRS-1 were investigated by Sanger automated sequencing in 12 BC and 14 CRC cell lines using an ABI PRISM^®^ 310 genetic analyzer (Applied Biosystems, Foster City, CA, USA). In 33 primary CRCs, the entire IRS-1 coding sequence, including intron-exon boundaries, was analyzed by DHPLC using the Wave^®^ nucleic acid fragment analysis system (Transgenomic, Inc., San Jose, CA, USA) and direct sequencing of the positive samples. The entire IRS-2 coding sequences was analyzed by direct sequencing in all BC and CRC samples. In the controls, the entire IRS-2 coding sequence was analyzed by single-strand conformation polymorphism (SSCP) technique and sequencing. Primers and polymerase chain reaction (PCR) conditions are detailed in Tables I and II. To exclude PCR artifacts, all mutations were confirmed on both DNA strands and in duplicate experiments on separately extracted DNA. Variant nomenclature followed human genome variation society guidelines (http://www.hgvs.org/mutnomen). The cDNA NM_005544.2 and protein NP_005535.1 sequences were used for IRS-1 reference sequence, and the cDNA NM_003749.2 and protein NP_003740.2 sequences for IRS-2 reference sequence. DNA +1 corresponds to the A of the ATG translation initiation codon. MSI analysis of 33 primary CRCs was performed as previously described (15). *In silico* analysis to assess likely pathogenicity of the variants was performed using PolyPhen (http://genetics.bwh.harvard.edu/pph/) and SIFT (http://sift.jcvi.org/www/SIFT_seq_submit2.html). SIFT scores were classified as intolerant (0.00–0.05), potentially intolerant (0.051–0.10), borderline (0.101–0.20), or tolerant (0.201–1.00) according to the classification proposed by Ng and Henicoff (18) and Xi *et al*(19).

### Phylogenetic conservation

Full length orthologous protein sequences from a range of animal species were extracted from GenBank. We confirmed these as orthologs based on database annotation of identity and/or predicted function, as well as on the requirement that the sequence be the top hit in a BLAST of the human sequence against the genome database for each organism. Human protein sequences were aligned to the following vertebrate orthologs: IRS-1, *Pan troglodytes*, *Macaca mulatta*, *Mus musculus*, *Rattus norvegicus*, *Canis lupus familiaris*, *Bos taurus*, *Sus scrofa*, *Equus caballus*, *Monodelphis domestica* and *Gallus gallus*; IRS-2, *Pan troglodytes*, *Mus musculus*, *Rattus norvegicus*. The computational analysis was carried out at the http://www.ebi.ac.uk/Tools/msa/clustalo/ website. We inferred that mutations were functional if occurring at residues completely conserved in orthologs.

Accession codes were as follows: GenBank mRNA: human IRS-1 NM_005544 (version NM_005544.2), IRS-1 ortholog protein accession numbers: NP_005535.1 *Homo sapiens*, XP_001134895.1 *Pan troglodytes*, XP_001109882.1 *Macaca mulatta*, NP_034700.2 *Mus musculus*, NP_037101.1 *Rattus norvegicus*, XP_543274.2 *Canis lupus familiaris*, XP_581382.2 *Bos taurus*, AAT99886.1 *Sus scrofa*, XP_001915510.1 *Equus caballus*, XP_001373872.1 *Monodelphis domestica*, NP_001026741.1 *Gallus gallus*. IRS-2 ortholog protein accession numbers were: NP_003740.2 *Homo sapiens*, XP_529580.2 *Pan troglodytes*, NP_001074681.1 *Mus musculus*, NP-001162104.1 *Rattus norvegicus*.

### Statistical analysis

Chi-square test 2-tailed was used to calculate all reported P-values using GraphPad v4 software (GraphPad Software, Inc., San Diego, CA, USA).

## Results

### IRS-1 and IRS-2 variants in BC

The complete coding region and intron/exon boundaries of IRS-1 were investigated by automated sequencing in 12 BC cell lines (BT-20, BT-474, EVSA/T, MCF10A, MCF7, MDA-MB-134, MDA-MB-231, MDA-MB-365, MDA-MB-453, SK-BR-3, T47D, HCC1937). Tables III and IV summarize the frequencies of 3 allelic variants identified in IRS-1. Two common variants, c.702G>C and c.2678G>C, were detected also in the general population (Table IV) (20–28). The novel amino acid substitution, p.Arg267Cys, identified in MDA-MB-365, occurs in the well-conserved PTB domain, and is probably damaging as determined by *in silico* analysis performed with PoliPhen and is predicted to affect protein function by SIFT. The p.Arg267Cys substitution has never been described in the general population and type 2 diabetes patients (20–28) and is not described in public databases.

The complete coding region and intron/exon boundaries of IRS-2 were investigated by automated sequencing in 11 BC cell lines. Table IV summarizes the frequencies of 6 allelic variants identified in IRS-2 (c.2169C>T, c.2448T>C, c.2487C>T, p.Gly879Ser, pGly882Ala and p.Gly1057Asp), that were also detected in the general population (17,28–30) and are described in public databases.

### IRS-1 and IRS-2 in CRC

The coding region and short intron-exon borders of IRS-1 were investigated by automated sequencing in 14 CRC cell lines and by DHPLC and automated sequencing in 33 primary CRCs. Tables V and VII lists the 21 allelic variants identified in IRS-1. The coding region and short intron-exon borders of IRS-2 were investigated by automated sequencing in 12 CRC cell lines and 33 primary CRCs. Tables VI and VII summarize the 18 distinct allelic variants identified in IRS-2. Some of the detected IRS-1 and IRS-2 variants are common polymorphisms also found in the general population (Table VII) (17,20–30) and are described in public databases.

The novel variants identified in CRC included 11 for IRS-1 (5 amino acid substitutions, 2 frameshifts, 1 splice mutation and 5 silent variants) and 7 for IRS-2 (2 insertions, 4 amino acid substitutions and 1 silent variant) (Tables V and VI). Several of these variants (9 for IRS-1: p.Gln655His, p.Asp1014Gly, p.Asp1181His, p.Pro1203Ser; p.Gly40fs, IVS1+4C>T, c.2766G>A, c.3168C>T, c.3618C>T; 4 for IRS-2: pPro710Ser, p.Asp782Asn, pVal798Ile, pGly1230Ser) were identified in CRC cell lines. One germline IRS-1 variant (pPro559Leu) and a somatic frameshift mutation (p.Gly597fs) were identified in primary CRC cases. These variants were not detected in the control subjects (0/47), despite the fact that IRS-1 has been extensively analyzed as a candidate gene for type 2 diabetes (20–27) and are not described in public databases. Two in-frame insertion mutations in IRS-2, one germline (p.Ala701_Val702insAla) and the other tumor-associated (p.Asn28_His29insAsn), were identified in CRC cases. These genetic variants were not detected in the control subjects of this study and in previous mutational analyses (17,28–30) and are not described in public databases. Overall, nearly 17% of the CRC tested (cell lines and primary CRC cases) had unique missense or a deletion or insertion mutations in IRS-1 and/or IRS-2. These variants are widely dispersed in the coding regions of IRS-1 and IRS-2, but most of the missense variants are predicted to substitute evolutionarily conserved amino acids (Tables III, V and VI).

### Microsatellite instability analysis

MSI status was assessed in 33 primary CRCs (15). Two of these showed an MSI-H phenotype. According to publicly available data [http://www.sanger.ac.uk/genetics/CGP/CellLines/ and references (31–33)] 5 of the 14 CRCs cell lines analyzed (DLD1, HCT15, HCT116, LoVo and MIP101) are MSI-H. The IRS-1 nucleotide deletions identified in LoVo (c.119delG) and in an MSI-H primary CRC (c.1791delG) occurred in the contest of coding mononucleotide repeats (5 and 8 G repeats, respectively). Therefore coding IRS-1 repeats could be a target of defective mismatch repair (MMR) in CRC. To test this hypothesis, we analyzed 60 additional CRCs with an MSI-H phenotype for G deletions in the 5 G (c.119delG) and 8 G (c.1791delG) repeats of IRS-1. No mutations were identified in the 5 G repeat, while 5 additional deletions occurred in the 8 G repeat (Table V). Overall deletions in the 8 G repeat of IRS-1 were detected in 9.0% (6/67) of the tested CRCs with an MSI-H phenotype.

### In silico analysis of missense variants

PolyPhen (available at http://genetics.bwh.harvard.edu/pph/) was used to predict possible impacts of amino acid substitutions on protein structure and function. Of the 5 novel amino acid substitutions in IRS-1, 2 (pPro559Leu and pPro1203Ser) were scored as probably damaging and 3 (pGln655His, pAsp1014Gly and pAsp1181His) as possibly damaging. Of the 4 novel amino acid substitutions in IRS-2, 2 (pAsp782Asn and pGly1230Ser) were scored as possibly damaging and 2 (pPro710Ser and pVal798Ile) as benign. In an attempt to evaluate the functional relevance of the novel IRS-1 and IRS-2 amino acid substitutions, we employed the SIFT tool. Support for functional significance of the genetic alterations identified in the present study was derived from the analysis of the extent of evolutionary conservation of the altered residues in 11 orthologous IRS-1 and 4 orthologous IRS-2 proteins. The computational analysis carried out at http://www.ebi.ac.uk/Tools/msa/clustalo/ revealed that 5 out of 5 IRS-1 amino acid substitutions occurred at amino acid residues which were evolutionary conserved in birds and mammals (Table V). Of the IRS-2 amino acid substitutions, 3 out of 4 were conserved in mammals and 1 was not conserved (Table VI).

## Discussion

Constitutive activation of IRS-1 has been found in various solid tumors, including BC (34). *In vivo* overexpression of IRS-1 and IRS-2 in the mammary gland of murine models was found to cause mammary tumorigenesis and metastasis (35), suggesting that IRS-1 and IRS-2 behave as oncogenes *in vivo*. The Gly972Arg IRS-1 polymorphism has been associated with increased BC risk for BRCA1 class II mutation carriers (10). In the present study, mutational analysis of IRS in BC and CRC identified several variants with pathogenic potential. In the BC cell line MDA-MB-365, we identified a novel variant of IRS-1, p.Arg267Cys. This mutation is located in the well-conserved PTB domain, shows a pathogenic potential by *in silico* analysis and was observed neither in our controls (1/24 vs. 0/94, P=0.046) nor in public databases. Although *in silico* analysis predicted a pathogenic potential for p.Arg267Cys, further *in vitro* and *in vivo* studies are necessary to assess the functional effect of this mutation. We identified genetic variants of IRS-2 in BC cell lines which were also detected in the general population suggesting that these are common polymorphisms.

It was shown that partial or absolute IRS-1 deficiency in mice carrying the APC^min/+^ mutation reduces intestinal tumorigenesis (13) and that IRS-1 is a β-catenin direct target gene (12). These data suggest that IRS-1 might be a regulator of the initiation of neoplastic transformation by β-catenin. Moreover, the G972R IRS-1 polymorphism has been significantly associated with CRC risk (9). We recently showed that IRS-1 is modulated according to CRC differentiation and we suggested a role for IRS-1 in CRC progression and metastatis (11). Therefore, IRS-1 protein may coordinate signaling pathways involved in CRC development and progression. We identified 11 novel genetic alterations of IRS-1 in CRCs. These mutations were not observed in our controls and were not present in public databases. Two frameshift mutations, c.1791delG and c.119delG, predicted to generate a truncated IRS-1 protein were respectively identified in the LoVo cell line and in a primary CRC, both showing an MSI-H phenotype. The mutations, both in heterozygosity, occurred in the context of 5 and 8 G repeats, respectively. The frequency of these 2 frameshifts was assessed in 67 CRCs with an MSI-H phenotype. The frameshift in the 8 G repeat (c.1791delG) recurred in 6/67 (9.0%) cases, while the frameshift in the 5 G repeat (c.119delG) was detected in 1/67 cases (1.5%). Therefore the 8 G mononucleotide repeat of IRS-1 is an MSI target in MSI-H CRCs. The functional effect of this recurring mutation is not known. It is possible that the truncation activates the oncogenic potential of IRS-1 (36), or alternately that the corresponding allele is inactivated and this also may contribute to the tumor biology. In this regard, we previously found that in mucinous and undifferentiated CRCs, IRS-1 expression was low or absent (11). Moreover, it was previously shown that degradation of IRS-1 in lung cancer cells generated PI3K hyperactivity (37). The novel nucleotide variants identified in the CRC cell lines (p.Gln655His, p.Asp1014Gly, p.Asp1181His, p.Pro1203Ser, IVS1+4C>T, c.2766G>A, c.3168C>T, c.3618C>T) could be germline or somatic or acquired in culture, and thus their role is difficult to assess based on the available data, although the missense variants were determined to be putatively pathogenic by *in silico* analysis. A novel missense variant (pPro559Leu) identified in a CRC patient was in heterozygosity in both colorectal mucosa and primary CRC, and therefore occurred in the germline. Overall, considering that we identified 11 novel IRS-1 variants in 21/94 alleles and none in the controls (0/94; P<0.0001), mutations in this gene appear to occur at a considerable frequency in CRC.

Overall the CRCs (cell lines and primary CRC cases) were enriched in the IRS-1 nucleotide variants compared to the BC cell lines. There were significant differences in the frequencies of novel IRS-1 variants among the two groups studied, (1/24 in BC vs. 21/94 in CRCs, P=0.021) suggesting an association between IRS-1 variants and CRC.

Several studies have been published concerning the role of IRS-2 in CRC. The G1057D IRS-2 polymorphism has been significantly associated with CRC risk (9). In a previous study (38), we showed that IRS-2 was significantly expressed in the intestinal epithelium, where it localizes at top crypt and is directly controlled by the caudal-related homeobox protein (CDX2). IRS-2 RNA increases with spontaneous differentiation in both HT29 and Caco-2 cells and is downregulated in tumors of Apc^Min/+^ mice and FAP patients, that serve as models for β-catenin-dependent intestinal tumorigenesis (38). Moreover, the IRS-2 gene was reported to be amplified in 3/146 CRCs (14). We detected novel IRS-2 variants associated with the CRC cell lines (pPro710Ser, p.Asp782Asn, pVal798Ile, pGly1230Ser) and we did not establish whether these are germline, somatic or were acquired in culture. However the p.Asp782Asn and pGly1230Ser IRS-2 missense variants showed a putative pathogenic role by *in silico* analysis. One novel germline variant (p.Ala701_Val702insAla) was identified in heterozygosity both in the colorectal mucosa and in the primary CRC of one patient. We also detected a tumor-associated mutation (p.Asn28_His29insAsn) in a primary CRC, but not in the matched mucosa.

In summary, we showed that IRS-1 and IRS-2 variants occur at a considerable frequency in CRC and BC. The novel mutations identified in the present study are predicted to affect protein function and thus may be involved in the modulation of functions relevant to breast and colorectal tumorigenesis. Further studies with *in vitro* and *in vivo* BC and CRC models are necessary to clarify the role of these mutations in tumor biology.

## Figures and Tables

**Table I:** List of primers used for polymerase chain reaction amplification of the *IRS-1* gene.

Forward primer (5′→3′)		Reverse primer (5′→3′)		TA (°C)
1S′-990:	TCTGCTCAGCGTTGGTGGT	1A-1815:	GCGGAACTCATCACTCATG	59
2S-1732:	ATGCAGGTGGATGACTCTG	2A-2686:	GCATCATCTCTGTGTACTCCTC	57
3S-2606:	GCACATCCCCTACCATTACC	3A-3330:	GGATCTTGGCAATGAGTAGTAGG	57
BS-3217:	ATGAACATGTCACCAGTGGG	BA-3838:	CCTCAGTGCCAGTCTCTTCC	58
BS3-3776:	CTTCTGTCAGGTGTCCATCC	4A3-4845:	CAGAGGCGAAGAACAGAATTC	59
1S′-990:	TCTGCTCAGCGTTGGTGGT	1A22-1557:	GACGTTCTTTGTCTGACCCAG	60
1S22-1491:	ACCCGCATTCAAAGAGGTC	1A-1815:	GCGGAACTCATCACTCATG	60
2S-1732:	ATGCAGGTGGATGACTCTG	2A2-2140:	AGCGGCTGTGGTTGAG	58
2S2-2071:	ACCAACAGAACCCACGC	2A-2686:	GCATCATCTCTGTGTACTCCTC	58
3S-2606:	GCACATCCCCTACCATTACC	3A2-2950:	GTGGGGCAGATACGCTC	59
3S2-2892:	TGGCCGAAAGGGCAGT	3A-3330:	GGATCTTGGCAATGAGTAGTAGG	59
BS-3217:	ATGAACATGTCACCAGTGGG	BA2-3565:	CAGCTGTGTCCACTTCTCG	60
BS2-3516:	CCACCATCAGGTTCTGCAG	BA-3838:	CCTCAGTGCCAGTCTCTTCC	60
BS3-3776:	CTTCTGTCAGGTGTCCATCC	4A-4191:	CGAGTGGGCAGCCAGCT	60
4S-4019:	GCTACGTGGACACCTCG	4A2-4461:	CTCAAAGGAAGCAGAGCTG	56
4S2-4425:	CGAGGATGTGAAACGCC	4A3-4845:	CAGAGGCGAAGAACAGAATTC	60

IRS, insulin receptor substrate.

**Table II:** List of primers used for polymerase chain reaction amplification of the *IRS-2* gene.

Forward primer (5′→3′)		Reverse primer (5′→3′)		TA (°C)
ATG 5S:	GCGCAAGGGTGGGAGGGAGC	A2:	CTCAGGGGGCTCCCAGCCA	68
B1:	CTGGAGGCCATGAAGGCGCTC	R:	GGCGAAGGCACTACAGGGTG	67
S:	AGGAGGAGCGTCTGGAGCCTC	T:	GTAGTCGGAGAGCGGAGACC	67
U:	TCGCTCTTGTCCGCCAGCAG	V3:	CATCTCGGTGTAGTCACCATTG	68
Z:	CCTCATCGTTGTCCTCGGAC	XX2:	AGTGGTGGGACAAGAAGTCA	62
K2:	CTTCCAGAATGGTCTCAACTAC	EX1:	GGCTTCTGGGTCAAGGT	62
EX2:	TGACCCAGGTCCTAGCTG	XX:	TGACATGTGCACATCCTGGTG	58
ATG 5S:	GCGCAAGGGTGGGAGGGAGC	BATG:	AGCCCTCCTGCTCCTGCTCG	66
C:	TCTACACCAAGGACGAGTACTTCG	D:	GATGTTCATGAGCTGCAGC	60
18UP:	GCTTCGTGAAGCTCAACTGCGAG	19DW:	CGACGATTGGCTCTTACTGCG	66
B1:	CTGGAGGCCATGAAGGCGCTC	A2:	CTCAGGGGGCTCCCAGCCA	71
G1:	AAGTGCAGCTCGTGCAGGG	cM:	AGGTCCTCTTGCGCAGCCCTC	69
CC:	TGGACGAGTACGGCTCCAG	N:	CCCTGGGCTGCAAGATCTGCTT	60
O:	GCAGGAGCGACGACTACATG	P:	GCCATCTGCATGCTCCATGG	60
Q:	GAGGACAGTGGGTACATGCG	R:	GGCGAAGGCACTACAGGGTG	60
Z:	CCTCATCGTTGTCCTCGGAC	W:	CGCTGCTTTTCCTGAGAGAGAC	62
X:	ACCCCAAGCGCCACAACTCGG	Y:	CTTGTCTCCCGGCTGAGGAAG	64

IRS, insulin receptor substrate.

**Table III:** Novel *IRS-1* variant in breast cancer.

Nucleotide variant	Amino acid change	Allele frequency in BC	Allele frequency in controls	P-value	PoliPhen/SIFT/phylogenetic conservation of IRS-1 wild-type residue	BC patient code
c.798C>T	p.Arg267Cys	1/24	0/94	0.047	Probably damaging/intolerant/complete	MDA-MB-365

IRS, insulin receptor substrate; BC, breast cancer.

**Table IV:** Common *IRS-1* and *IRS-2* variants in breast cancer.

Nucleotide variant	Amino acid change	Allele frequency in BC	Allele frequency in controls	P-value
IRS-1				
c.702G>A	p.(=)	1/24	8/94	NS
c.2678G>C	p.(=)	3/24	0/94	0.0005
IRS-2				
c.2169C>T	p.(=)	16/22	105/316	0.0002
c.2448T>C	p.(=)	11/22	105/296	NS
c.2487C>T	p.(=)	2/22	70/208	NS
c.2635G>A	p.Gly879Ser	1/22	3/200	NS
c.2645G>C	p.Gly882Ala	1/22	0/50	NS
c.3171G>A	p.Gly1057Asp	6/22	244/798	NS

IRS, insulin receptor substrate; BC, breast cancer; NS, not significant.

**Table V:** Novel *IRS-1* variants in colorectal cancer.

Nucleotide variant	Amino acid change	Allele frequency in CRC patients	Allele frequency in controls	P-value	PoliPhen/SIFT/phylogenetic conservation of IRS-1 wild-type residue	CRC patient code
c.119delG	p.Gly40fs	1/132	0/94	NS	-/-/disruptive	LoVo
c.1676C>T	p.Pro559Leu	1/94	0/94	NS	Probably damaging/potentially intolerant/complete	1685K, germline
c.1791delG	p.Gly597fs	6/132	0/94	0.036	-/-/disruptive	1708K, somatic
c.1965G>T	p.Gln655His	3/94	0/94	NS	Possibly damaging/intolerant/complete	DLD1, HCT-15, MIP101
c.2766G>A	p.(=)	1/94	0/94	NS	NA	CBS
c.3041A>G	p.Asp1014Gly	1/94	0/94	NS	Possibly damaging/intolerant/complete	DLD1
c.3168C>T	p.(=)	1/94	0/94	NS	NA	DLD1
c.3541G>C	p.Asp1181His	1/94	0/94	NS	Possibly damaging/potentially intolerant/complete	HCT-15
c.3607C>T	p.Pro1203Ser	1/94	0/94	NS	Problably damaging/intolerant/complete	SW480
c.3618C>T	p.(=)	2/94	0/94	NS	NA	Colo205, SW480
IVS1+4C>T	Intron	3/94	ND	ND	NA	DLD1, HCT-15, MIP101

IRS, insulin receptor substrate; CRC, colorectal cancer; NA, not applicable; ND, not determined; NS, not significant.

**Table VI:** Novel *IRS-2* variants in colorectal cancer.

Nucleotide variant	Amino acid change	Allele frequency in CRC patients	Allele frequency in controls	P-value	PoliPhen/SIFT/phylogenetic conservation of IRS-2 wild-type residue	CRC patient code
c.30C>G	p.(=)	1/90	0/50	NS	NA	1607K, germline
c.84_85insAAC	p.Asn28_His29 insAsn	1/90	0/396^a^	0.036	NA	1708K, somatic
c.2103_2104 insGCC	p.Ala701_Val702 insAla	1/90	0/396^a^	0.036	NA	1738K, germline
c.2128C>T	p.Pro710Ser	1/90	0/396^a^	0.036	Benign/tolerant/complete	MIP101
c.2344G>A	p.Asp782Asn	1/90	0/396^a^	0.036	Possiblydamaging/tolerant/complete	MIP101
c.2392G>A	p.Val798Ile	2/90	0/396^a^	0.003	Benign/tolerant/non conserved	DLD1, HCT-15
c.3688G>A	p.Gly1230Ser	3/90	0/396^a^	0.0003	Possibly damaging/intolerant/complete	DLD1, HCT-15, MIP101

a Controls including samples analyzed in our laboratory plus 173 controls from Bottomley *et al*(17).

IRS, insulin receptor substrate; CRC, colorectal cancer; NS, not significant; NA, not applicable.

**Table VII:** Common *IRS-1* and *IRS-2* variants in colorectal cancer.

Nucleotide variant	Amino acid change	Allele frequency in CRC patients	Allele frequency in controls	P-value
IRS-1				
c.270C>T	p.(=)	2/94	3/94	NS
c.702G>A	p.(=)	2/94	8/94	NS
c.2412A>G	p.(=)	12/94	12/94	NS
c.2452G>C	p.Gly818Arg	1/94	2/94	NS
c.2911G>A	p.Gly971Arg	3/94	8/94	NS
c.3056T>C	p.Ile1019Thr	1/94	0/94	NS
c.3334C>T	p.Arg1112Trp	1/94	0/94	NS
c.3388G>A	p.Gly1130Ser	1/94	0/94	NS
c.3489A>C	p.(=)	1/94	0/94	NS
c.3606C>T	p.(=)	1/94	0/94	NS
IRS-2				
c.2169C>T	p.(=)	45/90	105/316	0.004
c.2448T>C	p.(=)	53/90	105/296	<0.001
c.2487C>T	p.(=)	22/90	70/208	NS
c.2635G>A	p.Gly879Ser	1/90	3/546^a^	NS
c.2645G>C	p.Gly882Ala	1/90	0/396^a^	0.036
c.2673G>C	p.(=)	8/90	ND	ND
c.3093G>A	p.(=)	1/90	0/50	NS
c.3099A>G	p.(=)	27/90	ND	ND
c.3171G>A	p.Gly1057Asp	27/90	244/798	NS
c.3730T>C	p.Ser1244Pro	1/90	0/396^a^	0.036
c.3788G>T	p.Gly1263Val	1/90	0/396^a^	0.036

a Controls including samples analyzed in our laboratory plus 173 controls in Bottomley *et al*(17).

IRS, insulin receptor substrate; CRC, colorectal cancer; NS, not significant; ND, not determined.
